# Supplementary material for: The microbiome biomarkers of pregnant women’s vaginal area predict preterm prelabor rupture in Western China
Source: Front Cell Infect Microbiol. 2024 Oct 31;14:1471027. doi: 10.3389/fcimb.2024.1471027 (PMC11560878; doi:10.3389/fcimb.2024.1471027)
Supplement: Supplementary file 1 [file DataSheet1.zip › compare_1/Community/Pieplot/genus/group/C.genus.pieplot.pdf]

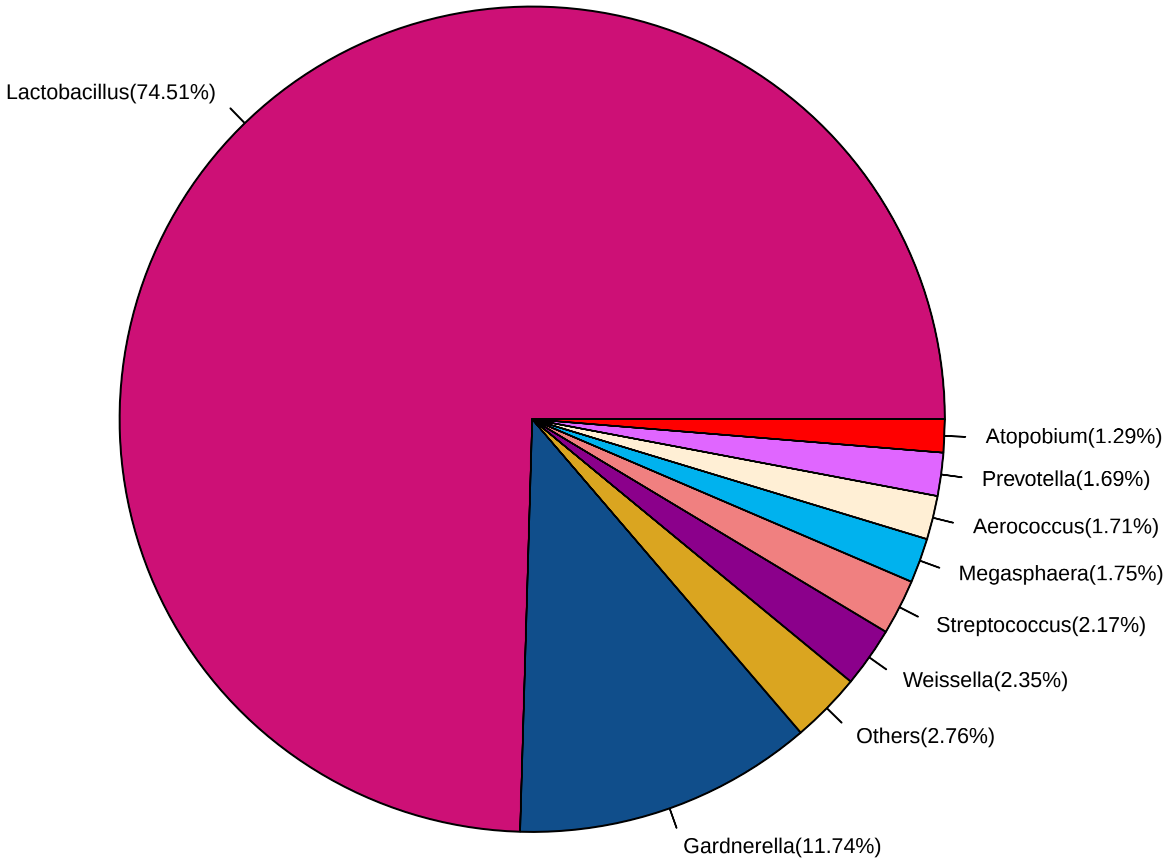
Lactobacillus(74.51%)
Atopobium(1.29%)
Prevotella(1.69%)
Aerococcus(1.71%)
Megasphaera(1.75%)
Streptococcus(2.17%)
Weissella(2.35%)
Others(2.76%)
Gardnerella(11.74%)
